# Zoophagic behaviour of anopheline mosquitoes in southwest Ethiopia: opportunity for malaria vector control

**DOI:** 10.1186/s13071-015-1264-9

**Published:** 2015-12-18

**Authors:** Fekadu Massebo, Meshesha Balkew, Teshome Gebre-Michael, Bernt Lindtjørn

**Affiliations:** Department of Biology, Arba Minch University, Arba Minch, Ethiopia and Centre for International Health, University of Bergen, Bergen, Norway; Aklilu Lemma Institute of Pathobiology, Addis Ababa University, Addis Ababa, Ethiopia; Centre for International Health, University of Bergen, Bergen, Norway

**Keywords:** *Anopheles arabiensis*, *Anopheles marshalli*, Bovine blood meal, Feeding preference, Human blood meal, Zoophagic vectors

## Abstract

**Background:**

Increased understanding of the feeding behaviours of malaria vectors is important to determine the frequency of human-vector contact and to implement effective vector control interventions. Here we assess the relative feeding preferences of *Anopheles* mosquitoes in relation to cattle and human host abundance in southwest Ethiopia.

**Methods:**

We collected female *Anopheles* mosquitoes bi-weekly using Centers for Disease Control and prevention (CDC) light traps, pyrethrum spray catches (PSCs) and by aspirating from artificial pit shelters, and determined mosquito blood meal origins using a direct enzyme-linked immunosorbent assay (ELISA).

**Results:**

Both *Anopheles arabiensis* Patton and *An. marshalli* (Theobald) showed preference of bovine blood meal over humans regardless of higher human population sizes. The relative feeding preference of *An. arabiensis* on bovine blood meal was 4.7 times higher than that of human blood. *Anopheles marshalli* was 6 times more likely to feed on bovine blood meal than humans. The HBI of *An. arabiensis* and *An. marshalli* significantly varied between the collection methods, whereas the bovine feeding patterns was not substantially influenced by collection methods. Even though the highest HBI of *An. arabiensis* and *An. marshalli* was from indoor CDC traps collections, a substantial number of *An. arabiensis* (65 %) and *An. marshalli* (63 %) had contact with cattle. *Anopheles arabiensis* (44 %) and *An. marshalli* (41 %) had clearly taken bovine blood meals outdoors, but they rested indoors.

**Conclusion:**

*Anopheles* mosquitoes are zoophagic and mainly feed on bovine blood meals than humans. Hence, it is important to consider treatment of cattle with appropriate insecticide to control the zoophagic malaria vectors in southwest Ethiopia. Systemic insecticides like ivermectin and its member eprinomectin could be investigated to control the pyrethroid insecticides resistant vectors.

## Background

In Ethiopia, *Anopheles arabiensis* Patton is responsible for malaria transmission [1, 2]. *Anopheles pharoensis* Theobald is the secondary vector [1]. *Anopheles amharicus* Hunt, Wilkerson & Coetzee [3]*,* previously known as *An. quadriannulatus* sp. B, is zoophagic and has no role in malaria transmission [4]. Currently, the roles of *An. funestus* Giles and *An. nili* (Theobald) are uncertain because the species are reported rarely and none of them were positive for *Plasmodium* species [2, 5]. *Anopheles coustani* Laveran, *An. marshalli* (Theobald) and *An. demeilloni* Evans were reported from south Ethiopia [6], but none of them were tested for blood meal origins and circumsporozoite proteins detection. A substantial proportion of *An. christyi* (Newstead & Carter)*, An. cinereus* Theobald and *An. demeilloni* had human blood meal origin in south-central highland of Ethiopia [7].

The tendency of malaria vectors to feed on humans (amplifying host of malaria) increases the chance of malaria transmission to the susceptible human hosts [8]. On the other hand, those mosquitoes feeding on non-human hosts are likely have a low role in malaria transmission [9]. The current malaria vector control tools such as indoor residual spraying (IRS) and long lasting insecticidal treated nets (LLINs) are targeting endophagic and endophilic malaria vectors [10]. The most anthropophagic and endophagic malaria vectors are can successfully controlled by the LLINs and IRS, whereas LLINs and IRS might have little impact on those species predominantly feed on cattle outdoors [11]. The transmission of malaria continues even in areas with high coverage of indoor-based interventions, due to those vectors feeding on animals and humans outdoors [11, 12], hence there is a need to target all the possible blood meals sources of zoophagic malaria vectors for successful control of the species [10, 12]. Zooprophylaxis is the diversion of vectors to animals or treatment of animals with appropriate insecticides as a supplementary intervention to control the zoophagic vectors [13]. Treatment of animals using toxic chemicals to kill the zoophagic vectors while feeding on animals may decrease the vector population and hence malaria transmission [10]. The impact of zooprophylaxis may however be further maximized by increasing the coverage of indoor-based interventions (LLINs and IRS) to push mosquitoes outdoors where animals are mostly kept, thereby suppressing the human blood meal source and reducing the level of infection in the local vector population [14, 15].

Understanding the blood feeding behaviour of the local *Anopheles* mosquitoes is important to determine the feeding preference of malaria vectors [16, 17], which can inform supplementary vector control interventions [10, 16, 17]. The objective of this study was to assess the relative feeding preferences of *Anopheles* mosquitoes in relation to cattle and human host abundance in Chano, Southwest Ethiopia.

## Methods

### Study area

This study was conducted in Chano village, 15 km north of Arba Minch town from May 2009 to April 2010. The village is located at 06°6.666' N and 37°35.775' E, at an altitude of 1206 m above sea level. Domestic animals are usually kept in compounds close to the houses at night and people usually sleep indoors throughout the year. There is no permanent or seasonal movement of animals in/out of the village for feeding or watering. Detailed information on the study area and collection methods have previously been published [18].

### Host surveys

The total number of human population in the study area was obtained from the epidemiological study conducted in the area during the same period [19]. The total number of cattle and other animals during the study period were obtained from the agricultural office in the village. In addition, during mosquito collections we recorded both the number of people in the houses, and number of cattle in the compounds where collections were made.

### Mosquito collections

Freshly fed *Anopheles* mosquitoes were collected bi-weekly for one year from May 2009 to April 2010. We used ten CDC light traps to collect indoor host-seeking *Anopheles* mosquitoes. The CDC light traps were hung 45 cm above the floor at the feet of sleeping persons who were protected by untreated nets. The light traps were turned on at 18:00 and off at 6:00 h by two trained field assistants in the community. On the following morning, the mosquitoes were transported to the entomology laboratory of Arba Minch University for species identification and preservation for blood meal analysis.

Indoor resting mosquitoes were sampled in the mornings (6:00 to 9:00 h) from 10 other randomly selected houses using the pyrethrum knockdown spray collection (PSC) technique following the recommendations of WHO [20]. Outdoor resting mosquitoes were collected in the mornings (6:30-10:30 h) from 10 pit shelters constructed according to the method of Silver [21], under the shade of mango trees in the compound of 10 selected houses. While collecting mosquitoes from pit shelters, the mouth of each pit shelter was covered by untreated bed nets to maximize collection by preventing mosquitoes from escaping.

### Mosquito processing

Female *Anopheles* mosquitoes were identified to species using morphological characteristics [22]. Abdomens were examined under a dissecting microscope and females classified into unfed, freshly fed, half-gravid and gravid [20]. Freshly fed *Anopheles* mosquitoes were preserved individually in vials containing dessicating silica gel for later blood meal analysis.

### Detection of blood meal sources

The blood meals of freshly fed *Anopheles* mosquitoes were analysed by a direct enzyme-linked immunosorbent assay [23] using human and bovine antibodies. Each blood meal sample was considered positive if the absorbance value exceeded the mean plus three times the standard deviation of four negative controls (laboratory colony of *An. arabiensis* not fed on blood). Positive controls contained human and bovine blood.

### Data analysis

The human blood index (HBI) and bovine blood index (BBI) were calculated as the proportion of mosquitoes fed on either human or bovine blood meals out of the total blood meals tested [17]. Mixed (human + bovine) blood meals were added to the number of human and bovine blood meals when calculating the HBI and BBI [24, 25].

A linear regression analysis was done to see the impact of cattle to human ratio and collection methods on human and bovine blood meal index of *Anopheles* mosquitoes. The relative feeding preference of *Anopheles* mosquitoes were calculated according to Hess et al. [26] by taking the percentage of freshly fed *Anopheles* mosquitoes with either humans or bovine blood meals divided by the percent of either human or cattle in the area.

The following assumptions were made to characterize the host feeding preference: 1) the abundances of people and cattle did not vary throughout the year, 2) there is no seasonal change in sleeping habits of people in the study area, 3) host defensive behaviour did not alter mosquito feeding success, 4) people and cattle available out of doors did not vary at different season. Data were entered and analysed using SPSS version 20 (SPSS Inc., Chicago. IL).

## Results

### Human and cattle population

The human population size was 6661, some three times higher than number of cattle (*n* = 2217). Goats, sheep, donkeys, and chickens were also present in the village.

### Blood meal origins of *Anopheles* mosquitoes

The blood meal origins of *Anopheles* mosquitoes are shown in Table 1. The higher proportion of *An. arabiensis* (58 %; 521 of 894), *An. marshalli* (64 %; 279 of 436) and *An. garnhami* (60 %; 21 of 35) from pit shelters had blood meals of bovine origin. *Anopheles arabiensis* (65 %; 644 of 988) and *An. marshalli* (63 %; 103 of 164) from CDC light traps had mixed blood meals of human and bovine origins. Only a low proportion of *An. arabiensis* (3 %; 27 of 894) and *An. marshalli* (3 %; 14 of 436) from pit shelters contained human blood. Some 44 % *An. arabiensis* and 41 % *An. marshalli* had bovine blood meals, but were found indoors in resting collections.

**Table 1:** Variation in blood meal origins of *Anopheles* mosquitoes from different collection sites in Chano village in southwest Ethiopia

			Blood meal origins			
Collection methods	Species	No. analysed	Human (%)	Bovine (%)	Mixed (%)	Unknown (%)
CDC light traps	*An. arabiensis*	988	94 (9.5)	70 (7.1)	644 (65.2)	180 (18.2)
	*An. marshalli*	164	45 (27.4)	6 (3.7)	103 (62.8)	10 (6.1)
	*An. garnhami*	7	4 (57)	0 (0.0)	2 (29)	1 (14)
	*An. pharoensis*	7	1 (14)	0 (0.0)	2 (29)	4 (57)
	*An. tenebrosus*	4	1 (25)	1 (25)	0 (0.0)	2 (50)
	*An. funestus group*	1	0 (0.0)	1 (100)	0 (0.0)	0 (0.0)
PSC	*An. arabiensis*	352	59 (16.8)	154 (43.8)	74 (21)	65 (18.4)
	*An. marshalli*	56	9 (16.1)	23 (41.1)	18 (32.1)	6 (10.7)
	*An. garnhami*	7	3 (42.9)	2 (28.6)	2 (28.6)	0 (0.0)
	*An. funestus group*	1	0 (0.0)	0 (0.0)	0 (0.0)	1 (100)
Pit shelters	*An. arabiensis*	894	27 (3.0)	521 (58.3)	89 (10)	257 (28.7)
	*An. marshalli*	436	14 (3.2)	279 (64)	54 (12.4)	89 (20.4)
	*An. garnhami*	35	2 (5.7)	21 (60)	5 (14.3)	7 (20)
	*An. funestus group*	14	0 (0.0)	5 (35.7)	3 (21.4)	6 (42.9)
	*An. tenebrosus*	1	0 (0.0)	0 (0.0)	0 (0.0)	1 (100)
Total		2967	259 (8.7)	1089 (36.7)	996 (33.6)	629 (21)

*PSC* pyrethrum spray catches, *CDC* centers for disease control and prevention

### Relative feeding preference of *Anopheles* mosquitoes

**Fig. 1:**
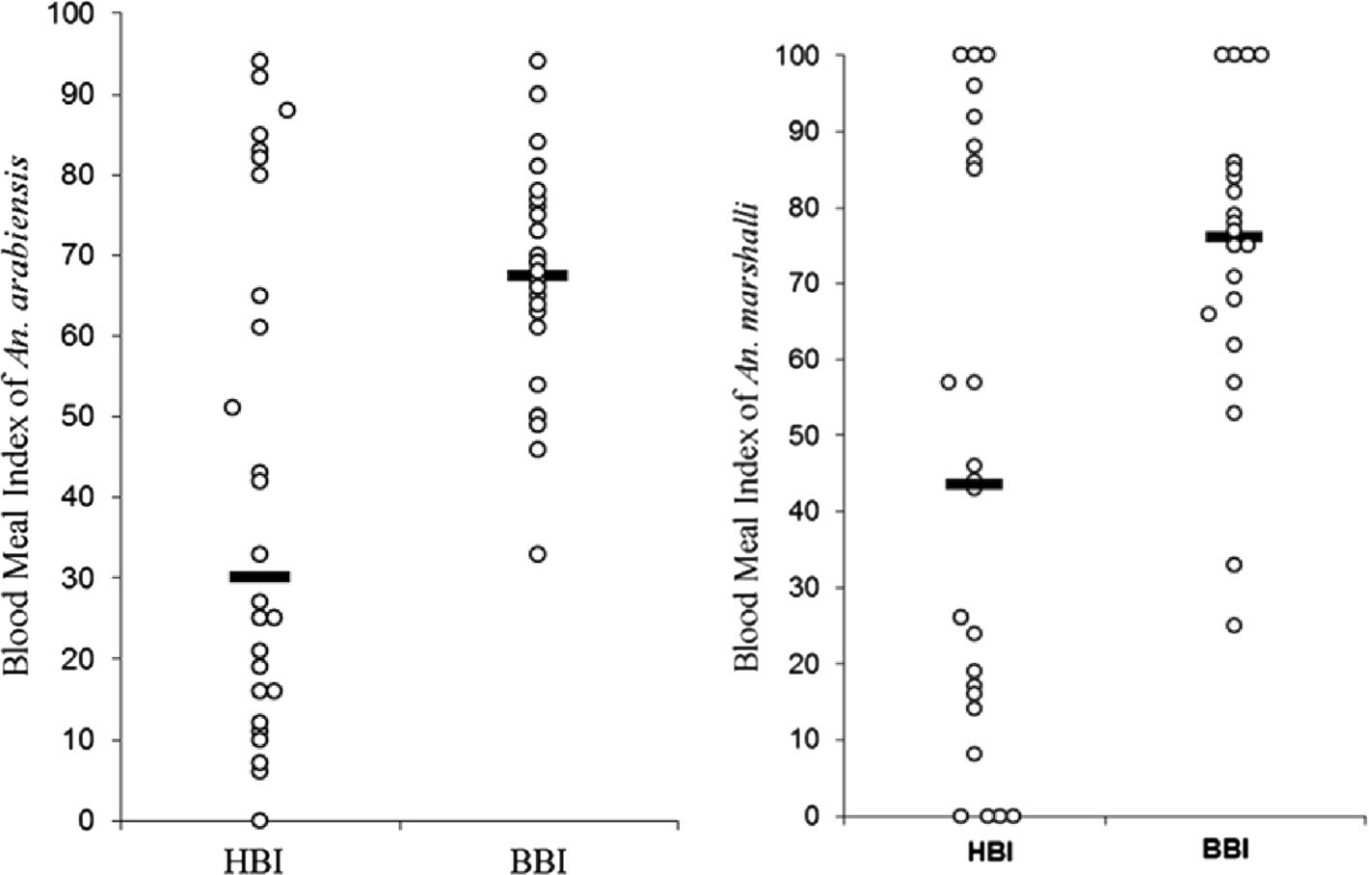
The human and bovine meal feeding preference of *Anopheles arabiensis* and *An. marshalli* from different sampling houses ( Median)

Regardless of the three-fold higher prevalence of humans in the study area, *An. arabiensis* and *An. marshalli* showed a strong preference of bovine blood meal over humans (Table 2 and Fig. 1). The relative feeding preference of *An. arabiensis* on cattle was 4.7 times higher than that on humans and *An. marshalli* was 6 times more likely to feed on cows than humans. The relative bovine blood meal feeding preference of *An. garnhami* was 5.3 times higher than humans. Thus, in this study area, *An. arabiensis, An. marshalli* and *An. garnhami* preferred bovine blood meals over humans.

**Table 2:** The relative feeding preference of *Anopheles* mosquitoes by considering human and cattle abundance from Chano village in southwest Ethiopia

Species	% HB	% HP	^a^FR	% BB	% BP	^b^FR
*An. arabiensis*	44	75	0.59	70	25	2.8
*An. marshalli*	37	75	0.49	74	25	2.9
*An. garnhami*	37	75	0.49	65	25	2.6
*An. funestus*	19	75	0.25	38	25	1.5
*An. pharoensis*	43	75	0.57	29	25	1.2
*An. tenebrosus*	20	75	0.27	20	25	0.8

*% HB* percent human blood meals, *% HP* percent human in populations, *% BB* percent bovine blood meals, *% BP* percent bovine, ^a^ *FR* forage ratios of human (% HB divided by % HP), ^b^ *FR*, forage ratios of cattle (% BB divided by % BP)

**Fig. 2:**
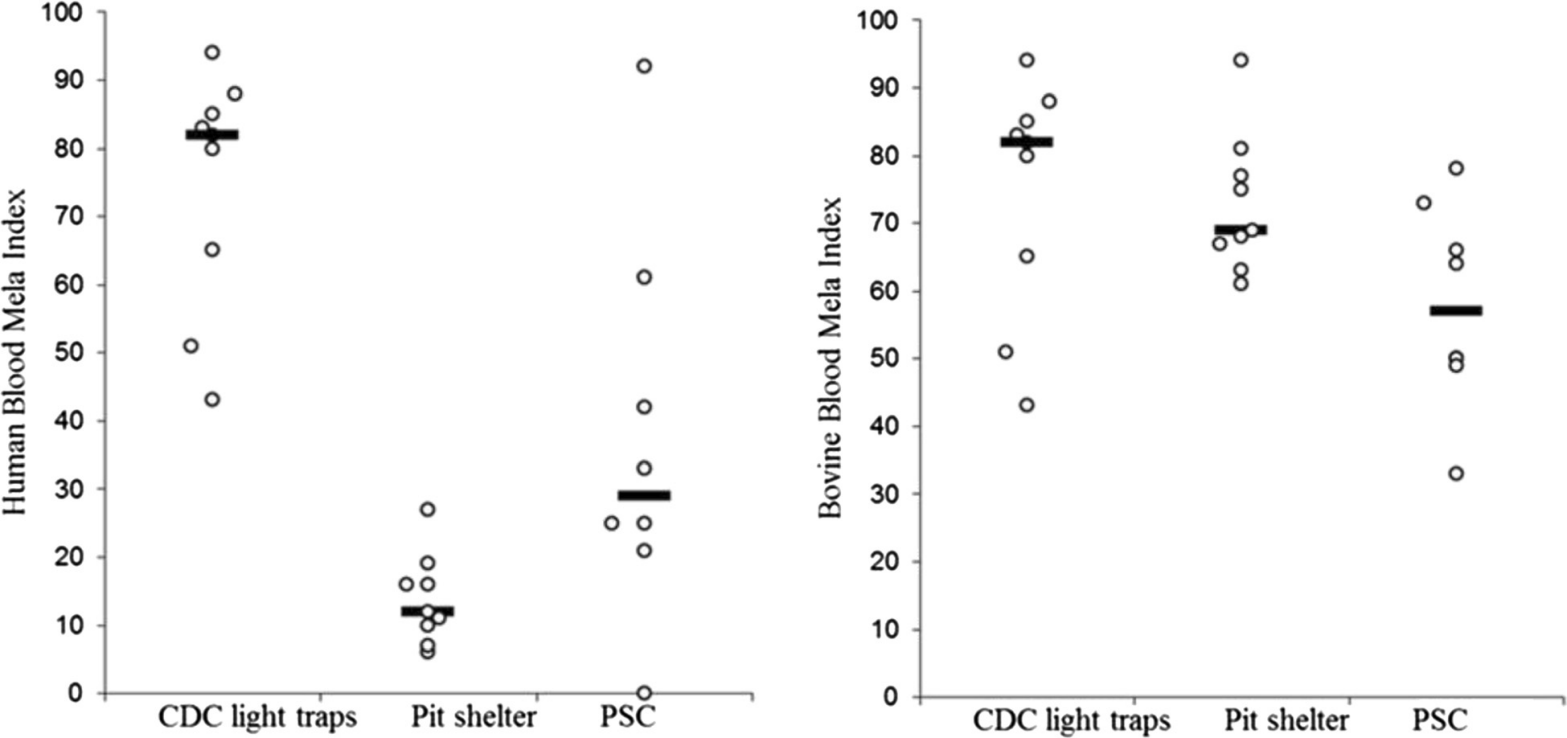
Human and bovine blood index of *Anopheles arabiensis* from different collection methods ( Median)

**Fig. 3:**
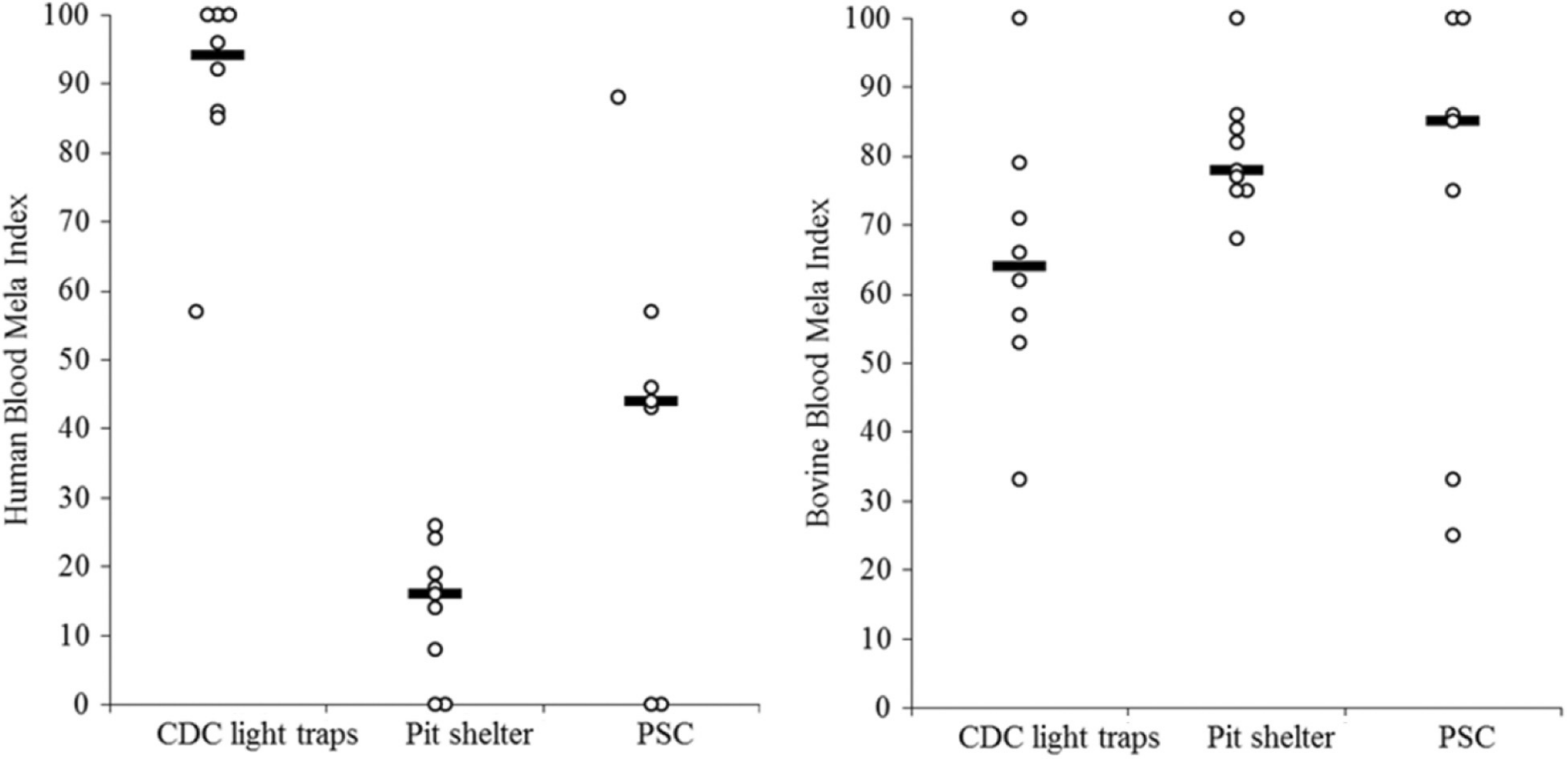
Human and bovine blood meal index of *Anopheles marshalli* from different collection methods ( Median)

The HBI of *An. arabiensis* significantly varied between the collection methods (*p* = 0.02), whereas the bovine feeding patterns of the species was not substantially influenced by collection methods (*p* = 0.17). The highest HBI of *An. arabiensis* and *An. marshalli* was from indoors CDC trap collections, while the lowest was from pit shelters (Figs. 2 and 3). *Anopheles arabiensis* showed a higher relative feeding preference on cattle and it remained higher in all collection methods. The feeding patterns of *An. arabiensis* and *An. marshalli* from PSC were inconsistent and showed variation between households (Figs. 2 and 3). Likewise, the human feeding patterns of *An. marshalli* (*p* = 0.005) varied between collection methods whereas the bovine feeding patterns of the species didn’t vary much by collection method (*p* = 0.86) and remained higher in all collection methods.

**Fig. 4:**
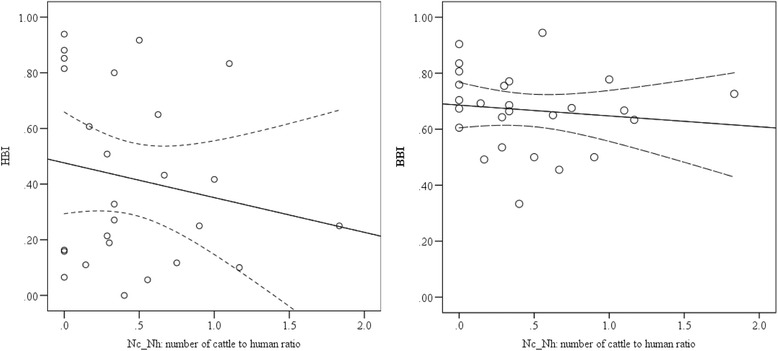
The relationship between human and bovine blood meal indices of *Anopheles arabiensis,* against the ratio of number of cows to humans in each household. The solid lines represent the linear analysis fit model and dash lines for the 95 % confidence intervals of the mean

**Fig. 5:**
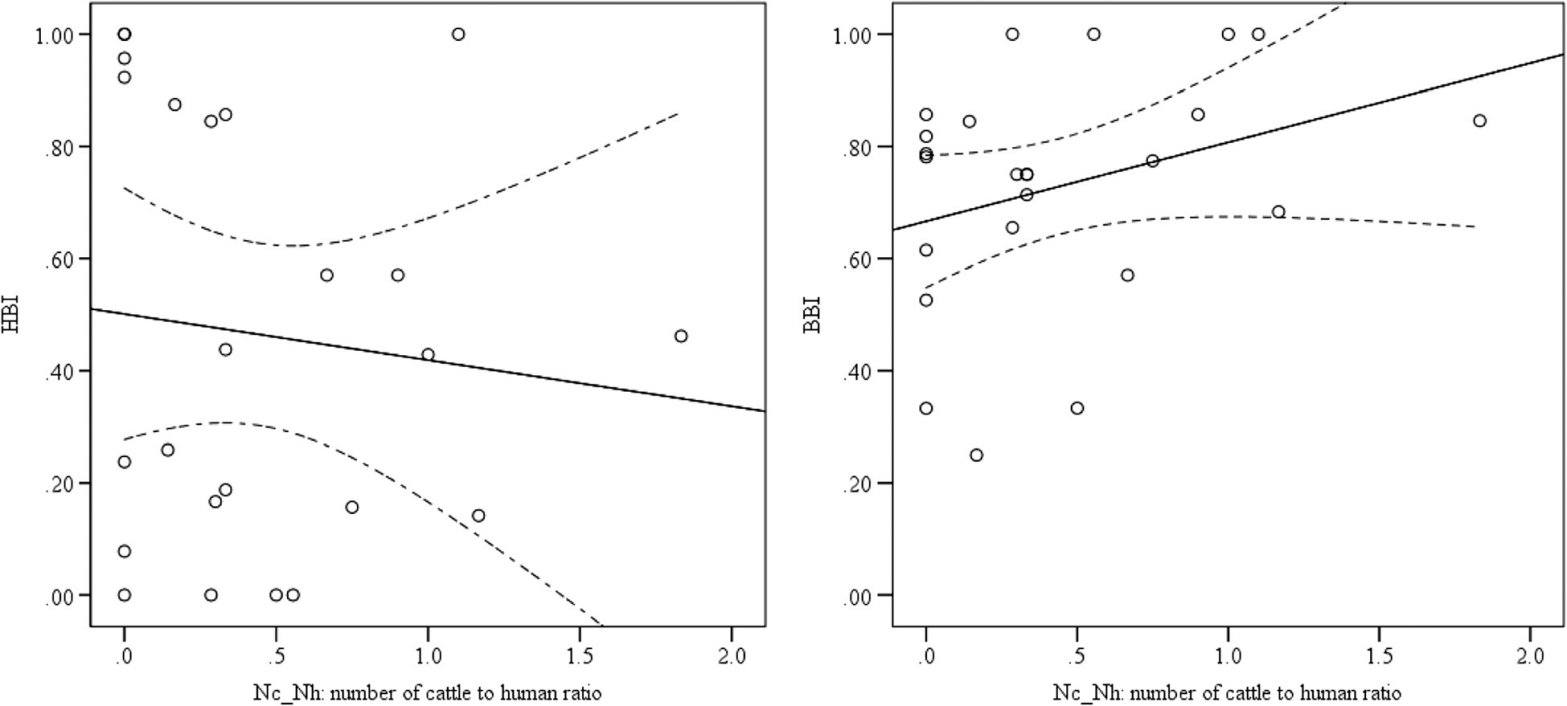
The relation between human and bovine blood meal indices of *Anopheles marshalli* against the ratio cattle to human in each household. The solid lines represent the linear analysis fit model and dash lines for the 95 % confidence intervals of the mean

The relative feeding pattern of both *An. arabiensis* and *An. marshalli* on humans decreased as the cattle to human ratio increased, whereas the cattle feeding preference either decreased for *An. arabiensis* or increased for *An. marshalli* as the cattle to human ratio increased (Figs. 4 and 5). The impact of cattle to human ratio of households on HBI (*p* = 0.87) and BBI (*p* = 0.86) of *An. arabiensis* was not significant. Similarly, the HBI (*p* = 0.59) and BBI (*p* = 0.18) of *An. marshalli* was not significantly influenced by the cattle to human ratio of households. This indicates that the human and bovine feeding patterns of *An. arabiensis* and *An. marshalli* slightly changed due to the number of cattle to human ratio of each household which in turn might be due to the accessibility of cattle outdoors in the village throughout the night.

**Fig. 6:**
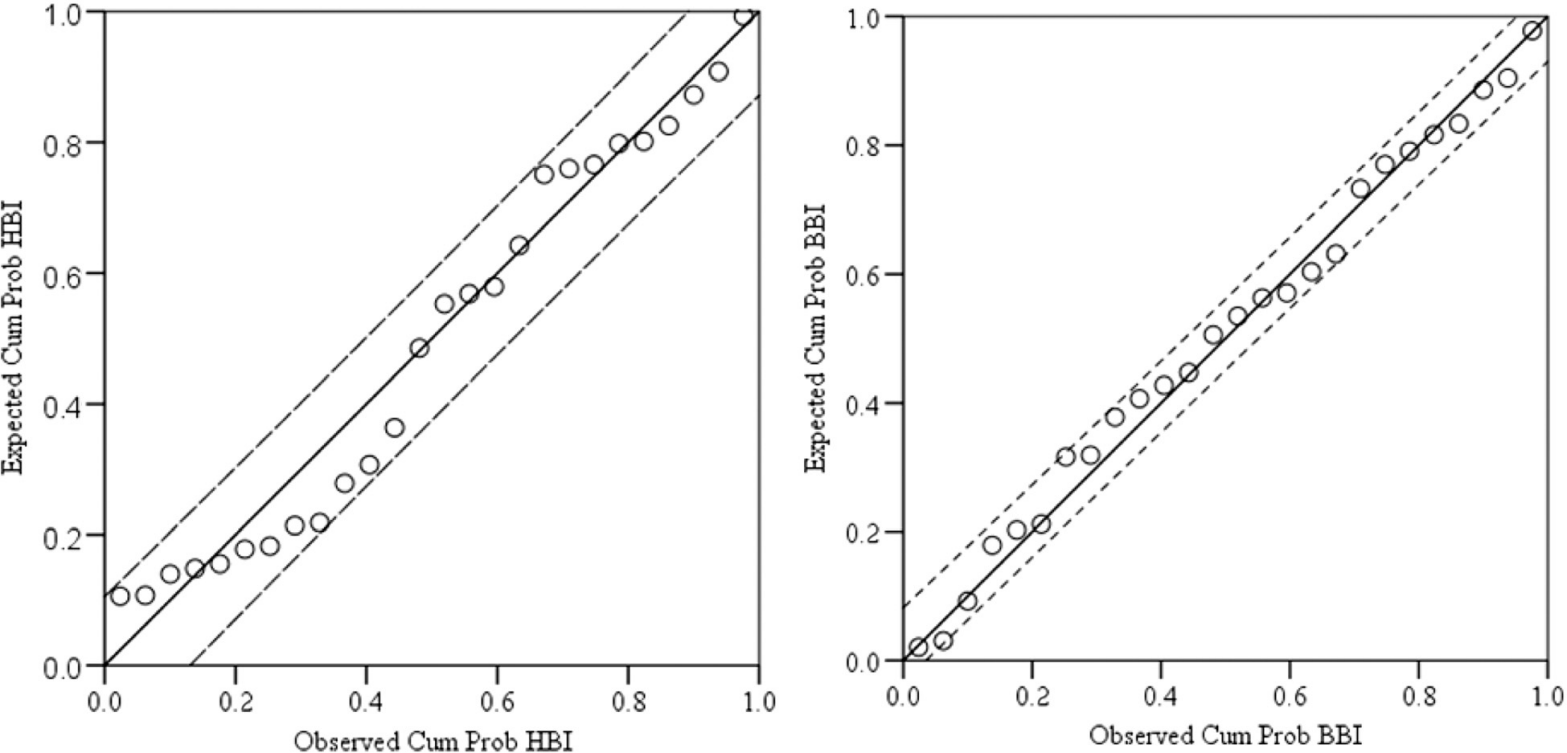
Comparison between observed and predicted human and bovine blood meal indices of *Anopheles arabiensis* using a linear regression analysis (n = 26, df = 24, *r* ^*2*^ = 0.97 for HBI and *r* ^*2*^ = 0.99 for BBI)

**Fig. 7:**
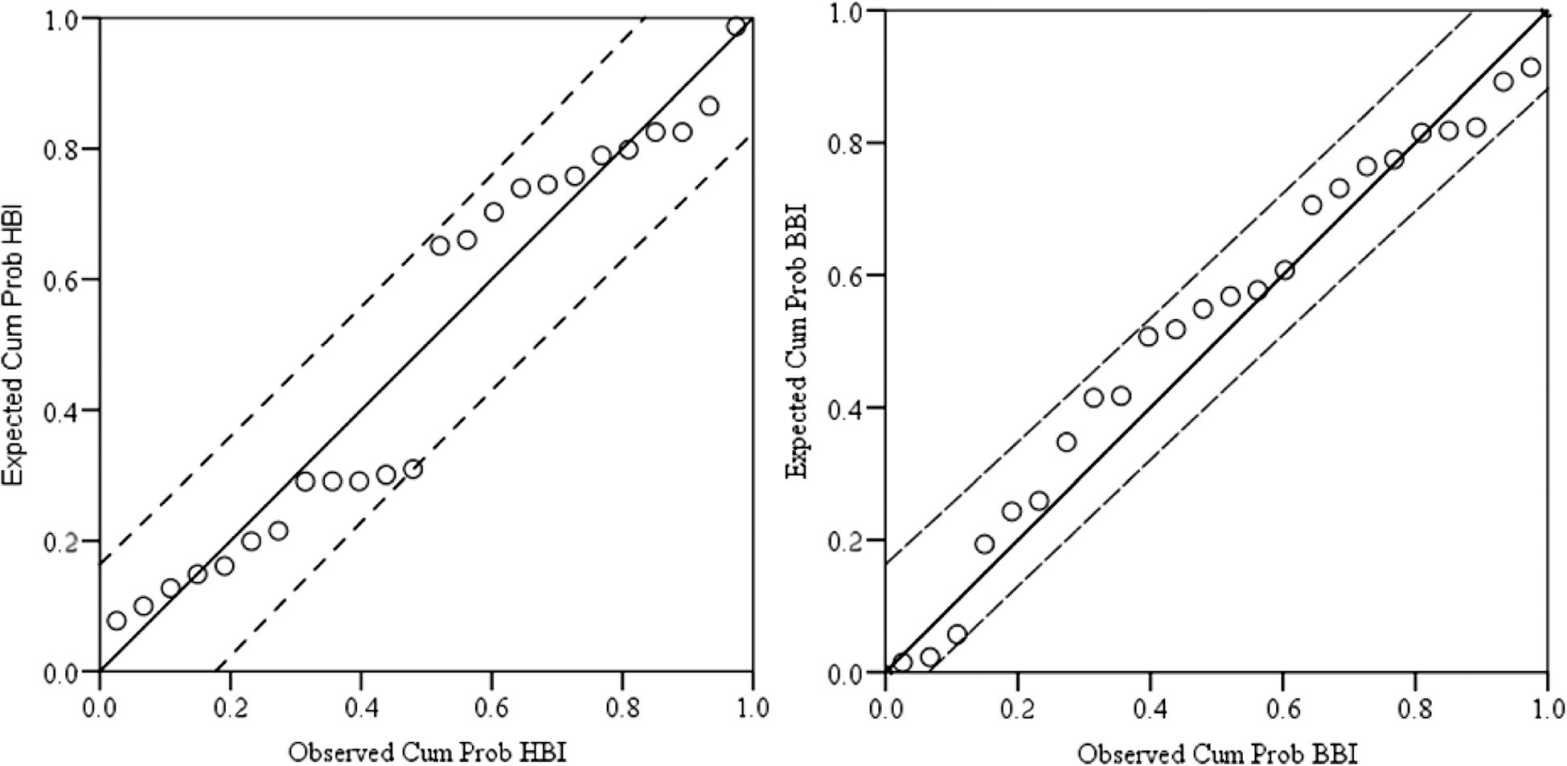
Comparison between observed and predicted human and bovine blood meal indices of *Anopheles marshalli* using a linear regression analysis (n = 24, df = 22, *r* ^*2*^ = 0.93 for HBI and *r* ^*2*^ = 0.96 for BBI)

The predicted and observed human and bovine blood meal indexes of *An. arabiensis* and *An. marshalli* were similar (Figs. 6 and 7) but the BBI fitted best for both species than HBI, indicating the bovine feeding pattern of the species is consistent in the area (Figs. 6 and 7).

## Discussion

*Anopheles* mosquitoes are zoophagic; mainly feeding on bovine blood meals than humans. We observed this in spite of the higher human proportion in the area. The relative feeding preferences of *An. arabiensis* and *An. marshalli* on human varied between collection methods with the highest human blood meal indexes from indoor CDC light traps collections. But, many of the human fed *An. arabiensis* and *An. marshalli* had contact with cattle since the higher human blood meal index was because of the mixed (human/bovine) blood meal origins. The bovine blood meal indexes of *An. arabiensis* and *An. marshalli* did not vary, and remained high at all collection methods indicating the consistency of bovine feeding patterns of the *Anopheles* mosquitoes in the village.

Our results are in agreement with the previous studies that reported the zoophilic feeding preferences of *An. arabiensis* [27–30], *An. marshalli* and *An. demeilloni* [16]. The feeding patterns of mosquitoes might be influenced by proximity, accessibility and defensive behaviours of hosts [18, 31]. In our study area, animals are usually kept outdoors at night where mosquitoes first encounter animals while searching for blood meal sources.

The higher relative feeding preference of *Anopheles* mosquitoes on cattle (zoophagic behaviour) can be considered as an opportunity to introduce supplementary vector control interventions based on zooprophylaxis - the diversion of mosquitoes from humans to animals [13, 14, 28]. Malaria vectors which mostly feed on human indoors can successfully be controlled by the LLINs and IRS, whereas those species predominantly feeding on cattle outdoors continue to transmit malaria regardless of high coverage of indoor based interventions [11]. Hence, there is a need to target those zoophagic species for control of human malaria [10, 12]. Zooprophylaxis can reduce malaria transmission by pulling mosquitoes toward the dead-end hosts so that the infectious mosquitoes effectively “waste” their sporozoites, and the susceptible mosquitoes cannot acquire parasitaemia from non-human hosts. The impact of zooprophylaxis can be further enhanced by increasing indoor interventions (e.g. bed nets) to protect humans from bites, thus, pushing mosquitoes outdoors towards the alternative mammalian blood sources [14] (dead-end host), effectively reducing infectious bites on humans [14]. In Ethiopia, keeping animals in separate sheds reduced the human biting rates of *An. arabiensis* showing that the animals had the capacity to pull mosquitoes [15]. In the same study, Seyoum et al. reported that sharing the house with animals increased the human biting rate of malaria vectors further supporting the pulling potential of animals [15].

Zooprophylaxis strategies can be further strengthened by treating cattle with insecticides (increasing the coverage of insecticides to all blood meal sources) to kill mosquitoes while feeding on animals, thus reducing the vector population and local malaria transmission [10, 13]. Spraying animals with pyrethroid insecticides reduced the incidence of malaria in Pakistan [13]. Habtewold et al. [32] identified two challenges while treating cattle to control *An. arabiensis:* one is the preference of *An. arabiensis* to feed on legs where insecticides washes off easily, and the second is short duration of the action of deltamethrin. Moreover, *An. arabiensis* in the study area is resistant to pyrethroid insecticides (the only class of insecticide recommended for spraying animals) including deltamethrin [18]. Alternative longer-lasting chemicals like ivermectin, a systemic insecticide widely used to control endoparasites and blood sucking ectoparasites of animals [33], may be used to control such zoophilic malaria vectors as *Anopheles* mosquitoes are sensitive to low concentrations of ivermectin [34].

The higher proportion of *An. arabiensis*, *An. marshalli* and *An. garnhami* that fed on human blood were from indoor host seeking collections which might be related with the low bed net use rate of the community during the study period [19], and to resistance of *An. arabiensis* to deltamethrin insecticide [18] or early biting behaviours of mosquitoes [35]. But, many mosquitoes had mixed (human/bovine) blood meal origins and had contact with cattle, suggesting that treatment of cattle with appropriate insecticides could be effective for controlling even those malaria vectors biting indoors. Those mosquitoes biting in the early hours of the night might be less affected by the indoor based interventions and more likely bite humans [36]. The role of *An. marshalli* and *An. garnhami* in malaria transmission need to be studied.

The higher bovine blood meal index from indoor resting collections shows the indoor resting preference of *Anopheles* mosquitoes after feeding on cattle outdoors. Thus, the existing indoor interventions such as LLINs and IRS are essential to reduce indoor transmission of malaria and also push mosquitoes out of houses [37]. A few *An. arabiensis*, *An. marshalli* and *An. garnhami* from pit shelters had human blood meals, and it is also important to consider these outdoor resting mosquitoes because they can maintain residual malaria transmission [38].

## Conclusion

In this study in southwest Ethiopia, *Anopheles* mosquitoes appeared preferentially zoophilic, feeding on cattle. It is important to consider treatment of cattle with appropriate insecticide to control the zoophilic malaria vectors in southwest Ethiopia. The possibility of using systemic insecticides like ivermectin needs to be considered to control the insecticide resistant malaria vectors in the area.
